# Comment on: Continuous renal replacement therapy with the adsorptive oXiris filter may be associated with the lower 28-day mortality in sepsis: a systematic review and meta-analysis

**DOI:** 10.1186/s13054-023-04588-2

**Published:** 2023-08-03

**Authors:** Xiaowei Huang, Fan Zhang, Yifei Zhong

**Affiliations:** 1 Department of Oncology, Longhua Hospital Shanghai University of Traditional Chinese Medicine, Shanghai, China; 2 Department of Nephrology, Longhua Hospital Shanghai University of Traditional Chinese Medicine, No. 725, Wanping South Road, Xuhui District, Shanghai, China

**Dear Editor,**

We read with interest the recent study by Wang et al. [1] on the association of continuous renal replacement therapy using adsorptive oXiris filters with 28-day mortality in sepsis, and we congratulate the authors on a comprehensive meta-analysis. Although this study has its strengths, several issues warrant further discussion.

First, for the primary outcome, 28-day mortality, the authors pooled data from randomized controlled trials and observational studies, which is contrary to the principle of pooling studies of similar design. In fact, combining data from these two types of study designs is only recommended when assessing harms/adverse effects [2]. Therefore, this result may mislead readers.

Second, based on the data extracted by the authors, we conducted a trial sequential analysis to explore the independent influence of random error on the estimation of intervention effects in meta-analyses and to avoid overestimating results by random error [3, 4]. According to alpha = 0.05 (bilateral), beta = 0.20 (power 80%), an expected relative risk reduction of 16.67%, and a control group event proportion of 60%, a required information size of 1278 was calculated. As shown in Fig. 1, the cumulative *Z*-curve did not cross trial sequential monitoring boundaries, and the sample size did not exceed the required information sizes, suggesting a lack of evidence that the oXiris filter, compared with other filters, reduced 28-day mortality significantly.

**Fig. 1:**
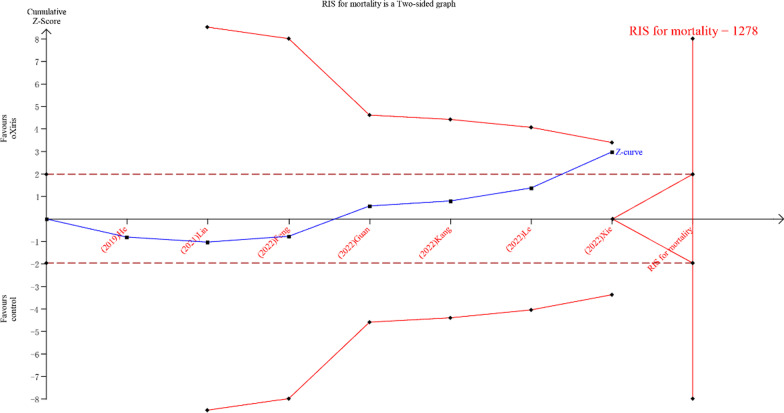
Trial sequential analysis of adsorptive oXiris filters vs. other filters for 28-mortality. RIS, required information sizes. The blue curve represents the *Z*-curve, the red curves above and below represent trial sequential monitoring boundaries, the dashed red dotted line represents the traditional level of statistical significance, and the red vertical line represents RIS value; the red lines on the sides closest to the horizontal line are boundaries for futility

Third, as the authors note, almost (93%) of the included studies originated from China, and most of these studies reported positive outcomes but lacked detailed reporting, making it difficult to highlight and replicate the critical factors behind treatment success. In addition, the lack of clear reporting leads to unclear risks of allocation concealment and selective reporting, which may jeopardize the internal validity of the meta-analysis results.

Given the limitations described above, the results that continuous renal replacement therapy for sepsis using adsorbent oXiris filters can be beneficial may be overstated, and therefore, readers should be cautious in interpreting the results of this vital meta-analysis.

## Data Availability

Not applicable.
